# Mechanisms of Intravascular Linear Ablation Induced Restenosis in Rabbit Abdominal Aorta

**DOI:** 10.1155/2018/7459276

**Published:** 2018-12-23

**Authors:** Qiang Chen, Manman Wang, Shuai Shao, Hongze Liu, Xiaodong Xia, Gary Tse, Meng Yuan, Yue Zhang, Xue Liang, Tong Liu, Guangping Li

**Affiliations:** ^1^Tianjin Key Laboratory of Ionic-Molecular Function of Cardiovascular Disease, Department of Cardiology, Tianjin Institute of Cardiology, Second Hospital of Tianjin Medical University, Tianjin 300211, China; ^2^Department of Medicine and Therapeutics, Chinese University of Hong Kong, Hong Kong; ^3^Li Ka Shing Institute of Health Sciences, Faculty of Medicine, Chinese University of Hong Kong, Hong Kong

## Abstract

**Objectives:**

Percutaneous coronary intervention (PCI) is the mainstay treatment for coronary artery disease but complications such as in-stent restenosis and thrombosis remain problematic. Radiofrequency balloon angioplasty (RBA) can improve lumen dimension, fusing intimal tears, and artery dissection but is associated with higher restenosis rate.

**Methods:**

After establishing an atherosclerosis model based on endothelial abrasion and high cholesterol diet, forty-five rabbits were randomly divided into three groups: RBA (n=20), percutaneous transluminal angioplasty (PTA) (n=20), and control groups (n=5). The RBA and PTA groups were subdivided according to harvested time posttreatment, respectively (1 hour, 7 days, 14 days, and 28 days). Aorta segments were then isolated for hematoxylin and eosin staining, Masson trichrome staining, immunohistochemistry, and Western blot for TLR-4, NF-*κ*B, MCP-1, and VCAM-1expression.

**Results:**

At 28 days, intimal area was significantly lower in the RBA group compared to the PTA and control groups, whilst luminal and medial area were comparable in the RBA and PTA group but higher and lower than the control group, respectively. Expression of TLR-4, NF-*κ*B, MCP-1, and VCAM-1 showed no significant difference between RBA and PTA groups.

**Conclusions:**

RBA can depress the intimal hyperplasia and promote dilatation of the artery to greater extents than PTA at 28 days. However, this did not involve TLR-4 signaling pathway, which likely plays a negligible role in mediating restenosis. Reduction of intimal hyperplasia may be due to injury of ablation to the tunica media and inhibition of VSMC proliferation and migration.

## 1. Introduction

Atherosclerosis is a major cause of coronary artery disease, contributing significantly to morbidity and mortality. Percutaneous coronary intervention (PCI) is an effective treatment but complications such as in-stent restenosis and thrombosis remain the major unresolved clinical problems, thereby limiting the overall efficacy of PCI [1]. Thus, the search for alternative strategies is desirable. From* in vivo *studies, radiofrequency balloon angioplasty (RBA), which involves mechanical pressure of balloon angioplasty complemented by radiofrequency (RF) energy, has been reported to decrease restenosis compared with using balloon angioplasty alone [2–4]. Yet, clinical studies have shown that RBA contributed to high restenosis rate compared with conventional percutaneous transluminal angioplasty (PTA), leading to aggravation of atherosclerosis [5, 6].

Toll-like receptors (TLRs) are a class of type I transmembrane pattern recognition receptors responsible for sensing invading pathogens or endogenous damage signals, activating the innate and adaptive arms of the immune system [7]. Over the past years, TLR4 has been implicated in the pathogenesis of atherosclerosis [8–11]. Thus, TLR-4 activation leads to activation downstream signaling pathway that causes translocation of nuclear factor *κ*B (NF-*κ*B) to the nucleus and promotes the transcription of proinflammatory cytokines [9, 12, 13]. NF-*κ*B activation has been found within human atherosclerotic lesions or after angioplasty, but not in normal arteries [14, 15]. The excessive vascular injury caused by superposition of thermal and balloon pressure in RBA may aggravate the inflammatory injury signal, leading to activation of TLR-4 signaling pathway and in turn contributing to higher degrees of restenosis. Previously, it has been speculated that RBA at a low temperature can reduce inflammation and in turn restenosis.

Currently two types of radiofrequency balloon catheters are available. The first employs RF electrode to heat the balloon fluid to required temperatures, which can be used to burn the tissue [3, 6]. The other uses a thin gold foil electrode adhered on the balloon surface which can be used to ablate the tissue directly [2]. Both techniques heat the blood vessel on circumferentially, which may contribute to cicatricial contracture could worsen vascular restenosis. We have thus designed a new radiofrequency balloon catheter which directly ablated the blood vessel in a linear fashion, potentially reducing lesion size and restenosis. In this study, we evaluated the effects of RBA with a new designed radiofrequency balloon catheter and compared these to those of conventional percutaneous transluminal angioplasty (PTA) in a rabbit atherosclerosis model. We also evaluate the degree of inflammatory injury by TLR-4 signaling pathway to address possible mechanisms underlying restenosis.

## 2. Material and Methods

### 2.1. Design of Radiofrequency Balloon Catheter

**Figure 1:**
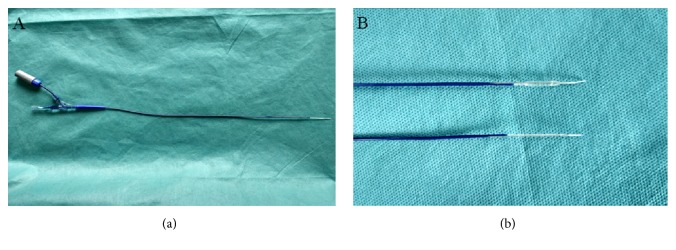
(a) The prototype of radiofrequency balloon catheter; (b) the tip of the radiofrequency balloon catheter.

The radiofrequency balloon catheter consists of a RF wire, temperature-control component, a 2cm polyester balloon, and catheter shaft (Figure 1). At the tip of catheter, a metal wire is parallel to the balloon. When the balloon is inflated and RF energy is delivered, the wire is compressed into the tissue and ablates the vessel in a linear manner.

### 2.2. Study Design and Preparation of the Rabbit Atherosclerosis Model

**Figure 2:**
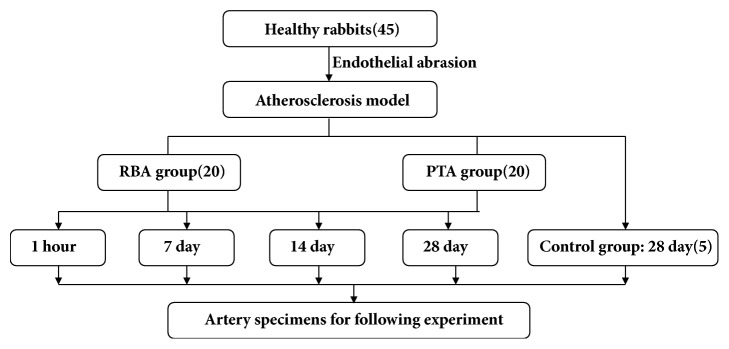
The flow chart of study groups.

Forty-five healthy rabbits (2.5–3.0 kg at the beginning of the study) were obtained from Beijing Medical Animals Research Institute. Experimental animal application approvals were obtained from the Experimental Animal Administration Committee of Tianjin Medical University and Tianjin Municipal Commission for Experimental Animal Control. A model of atherosclerosis was created by endothelial abrasion and high cholesterol diet for 6-8 weeks in 45 rabbits. According to the random number table method, 20 rabbits were defined as RBA group, and the rabbits were randomly divided into 4 subgroups according to harvested time posttreatment, respectively, five rabbits in each subgroup (1 hour, 7 days, 14 days, and 28 days). 20 rabbits were defined as PTA group, and the rabbits were also randomly divided into 4 subgroups according to harvested time post treatment, five rabbits in each subgroup. 5 rabbits were defined as control group, and rabbits were fed ordinary diet until 28 days after atherosclerosis was established (Figure 2).

### 2.3. Experimental Procedure

The rabbits were anesthetized by intravenous injection of 3% pentobarbital (1ml/kg). Under sterile conditions after one side femoral artery was isolated and an arched incision was made, a coronary balloon catheter (3.5*∗*15 mm) was used to induce endothelial abrasion in the abdominal aorta. Local hemostasis was performed by ligation. Animals were then placed on a high cholesterol diet for 6-8 weeks. After atherosclerosis model accomplished, RBA or PTA was performed in the rabbits from the contralateral femoral artery. When a femoral arteriotomy was performed, a 0.014-inch guide wire to the artery was used. Thereafter, the radiofrequency balloon catheter (3.5*∗*20 mm) or coronary balloon catheter (3.5*∗*20 mm) was inserted into a depth of 16 cm. Then the balloon inflations with RF were applied at balloon pressure of 8 atm, upper limit temperature of 55°C, inflation time of 20 s, and upper power limit of 30 Watts. The repeated inflation with heating in the same segment was applied after 180° rotation of the catheter and then we pulled back 20 mm of the catheter and repeated the procedure for 3 times from proximal to distal side of the artery. The PTA used by coronary balloon catheter (balloon pressure at 8 atm and inflation time of 20 s) was performed 4 times from proximal to distal side of the artery. Finally, rabbits were fed with ordinary diet and abdominal aorta artery were removed and preserved at 1 hour, 7 days, 14 days, and 28 days after treatment. Rabbits of control group were fed with ordinary diet until 28 days after the atherosclerosis model accomplished and abdominal aorta artery were removed and reserved.

### 2.4. Blood Sample Extraction

Blood samples were obtained from the auricular vein. The serum was prepared within 1 h by centrifugation at 1500g at room temperature for 15 min and stored at −80°C until needed for the assays. The serum and tissue concentrations of TLR4, NF-*κ*B, MCP-1, and VCAM-1 were measured by using commercial sandwich ELISA kits.

### 2.5. Histology

The rabbits were sacrificed at 1 hour, 7 days, 14 days, and 28 days after treatment. The abdominal aorta was removed and three segments at 1 cm intervals were excised. They were subsequently fixed in 4% paraformaldehyde solution for histological analysis. The tissues were dehydrated in graded alcohol and embedded in paraffin. Deparaffinized slides were stained with Haematoxylin and eosin (H&E) and Masson trichrome. For immunohistochemistry, arterial segments were fixed in 4% formalin and embedded in paraffin. The following primary antibodies were used: TLR-4 (1:500, ab22048), NF-KB P65 (1:500, ab-86299, abcam), MCP-1 (1:500, ab-25124, abcam), and VCAM-1 (1:2000, ab-98954, abcam). The following areas were manually identified and measured by computer-assisted analysis to determine vessel size (area circumscribed by the external elastic lamina area), medial area (area between internal and external elastic lamina), intimal area (area between the lumen and the internal elastic lamina), and luminal area (area circumscribed by the intimal/neointimal-luminal border). For each parameter, the mean value of three arterial segments was calculated.

### 2.6. Western Blot Analysis

Proteins were extracted using the total protein extraction buffer with complete, mini-, EDTA-free protease inhibitor cocktail tablets (Roche). Equal amount of proteins (20 *μ*g) was separated by denaturing discontinuous polyacrylamide gel electrophoresis (SDS-PAGE) and incubated with the specific primary antibody overnight at 4°C. The primary antibodies were purchased as follows: anti-GAPDH antibody (1:500, Hangzhou Xianzhi Inc., China), anti-NF-KB P65 antibody (1:4000, ab-86299, abcam), anti-MCP-1 (1:4000, ab-25124, abcam), and anti-VCAM-1 (1:2000, ab-98954, abcam). After washing, the membranes were subsequently incubated with the secondary antibody conjugated to horseradish peroxidase (HRP). Protein was visualized using enhanced chemiluminescence detecting kit (WBKLS0500, Millipore Corporation). The resulting bands were quantified using GeneTools software (Gene, USA).

### 2.7. RNA Isolation and RT-PCR

After removal of the arterial segments, the specimens were rapidly frozen in liquid nitrogen and stored separately at -80°C for investigating the messenger RNA (mRNA) expression of TLR-4, NF-*κ*B, MCP-1, and VCAM-1. Arterial segments were homogenized by Trizol reagent extracted with chloroform and precipitated in isopropyl alcohol. Specific oligonucleotide primer pairs were designed according to the sequences of GeneBank. The following PCR primers were used: TCGTGGATGACCTTGGCC/GATGCTGGTGCCGAGTAC (GAPDH); ACACCTCTTGACCTCACTCG/TCTTCCTGTACGTGTCCACC (NF-*κ*B); CCTCCAGCATGAAGGTCT/CATTCATGATCCTTGAAAGAAC (MCP-1); TGAGCGGGAAGGTGAGGAGTGAGG/CAGGATGGAGGAAGGGCTGACCAA (VCAM-1).

Commercially available kits (TaKaRa, Shiga, Japan) were used for RT-PCR. GAPDH were kept as control. The PCR consisted of 35 cycles of 94°C for 30s, 55°C (TLR-4, NF-*κ*B, MCP-1, and VCAM-1), 55°C (GAPDH) for 30s, and 72°C for 30s. The product was analyzed by 1% agarosegel electrophoresis. GAPDH, TLR-4, NF-*κ*B, MCP-1, and VCAM-1 mRNA expression was calculated by the 2^-△△Ct^ method using the Sequence Detection System 2.1 software (Applied Biosystems). The relative TLR-4, NF-*κ*B, MCP-1, and VCAM-1 mRNA expression was normalized to the GAPDH expression in the samples.

### 2.8. Statistical Analysis

All data were expressed as mean ± SD. Statistical analysis assuming normal distribution and variance was made. Subsequently, all significant data were analyzed with analysis of variance (ANOVA) and multiple comparisons were made using Tukey's multiple comparisons test. A *P* < 0.05 was considered statistically significant.

## 3. Results

### 3.1. Safety of Radiofrequency Balloon Angioplasty

One rabbit died from arteriorrhexis at 12 days post RBA. The abdominal aorta was removed and preserved for 14 days. In three rabbits of the RBA group, tissue adherence and fusion between the artery and the connective tissue of abdominal cavity was observed. The RF power used in four rabbits ranged from 22 to 28 Watts, which led to excessive ablation permeating through adventitia. Further study needs to be verified the optimum power of RBA without serious impairment.

### 3.2. Radiofrequency Balloon Angioplasty Produces Less Intimal Hyperplasia and Similar Increase in Vessel Size but Less Medial Thickening Compared to Percutaneous Transluminal Angioplasty

**Figure 3:**
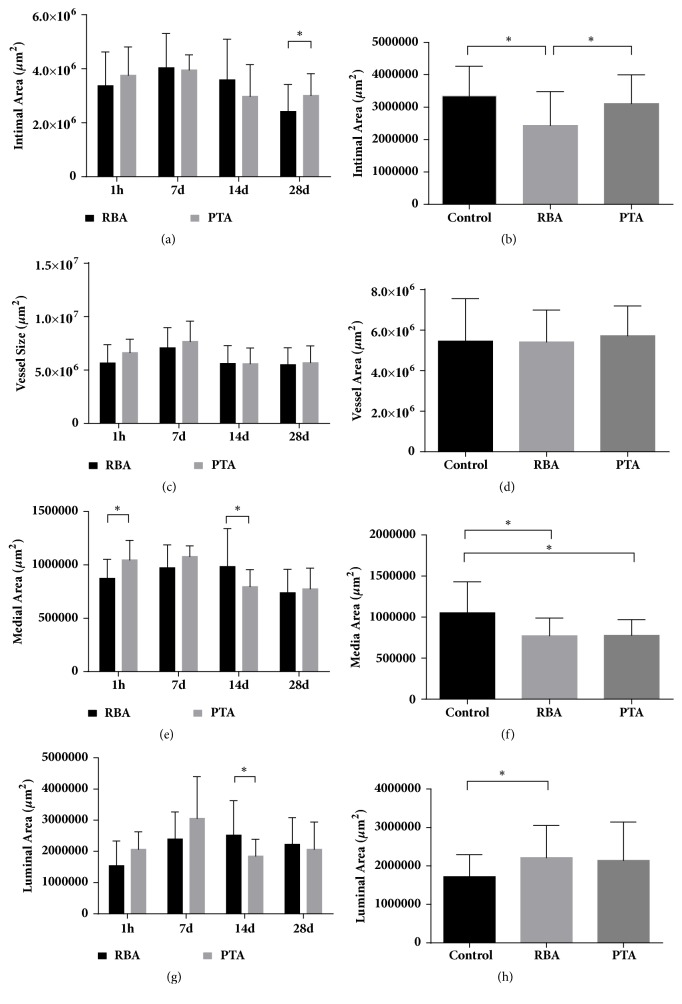
Vessel size, medial area, intimal area, and luminal area in each group (n=5 in each group; *∗P*<0.05).

Foam cells and high collagen content plaques were observed in both the RBA and PTA groups. The intimal area for these groups is shown in Figure 3(a). It showed a progressive time-dependent increase until 7 days and decreased to a minimum value 28 days for both groups. Compared with PTA and control group, radiofrequency balloon angioplasty produces less intimal hyperplasia compared to percutaneous transluminal angioplasty at 28 days (*P*<0.05; Figure 3(b), Table 1), in keeping with previous findings [16, 17].

The vessel size for these groups is shown in Figure 3(c). An increase in vessel size peaking at 7 days, decreasing after this time point for both RBA and PTA groups with no significant difference between them. No difference between these groups when compared to controls was observed (Figure 3(d), Table 1).

The medial area is shown in Figure 3(e). Interestingly, we found that when compared to the PTA group, the RBA group showed lower median area at 1 hour but larger area at 14 days (*p*<0.05). In both groups, the medial area was lower than that of the control group (Figure 3(f)). Finally, the luminal area in each group is shown in Figure 3(g). This was comparable between the RBA and the PTA groups at all timepoints except for 14 days, where the area was larger in the RBA group (*P*<0.05). The luminal area in both groups was significantly larger than that of controls.

**Figure 4:**
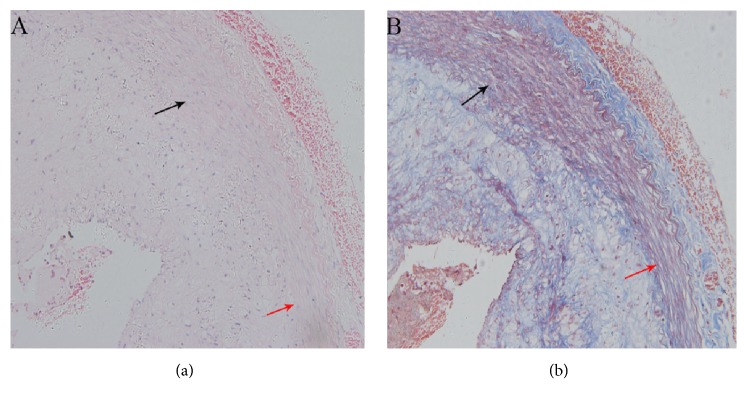
The specimen post-RBA demonstrated straightening and compression of elastic tissue in the medial at the injure site (red row) and no alteration of elastic tissue at the normal site (black row) ((a) H&E stain; (b) Masson trichrome stain).

Representative histological images are shown in Figure 4. The specimen from the RBA demonstrated straightening and compression of elastic tissue in the medial aspect of the injury site. The cell content was decreased and this was associated with increase in collagen fibers. The intimal aspect of the injury site did not appear to be affected. The medial area of RBA and PTA were statistically indistinguishable and significantly lower than control group at 28 days (*P*<0.05; Table 1).

### 3.3. TLR-4 Signaling Pathway Activation Did Not Differ between Radiofrequency Balloon Angioplasty and Percutaneous Transluminal Angioplasty Groups

**Figure 5:**
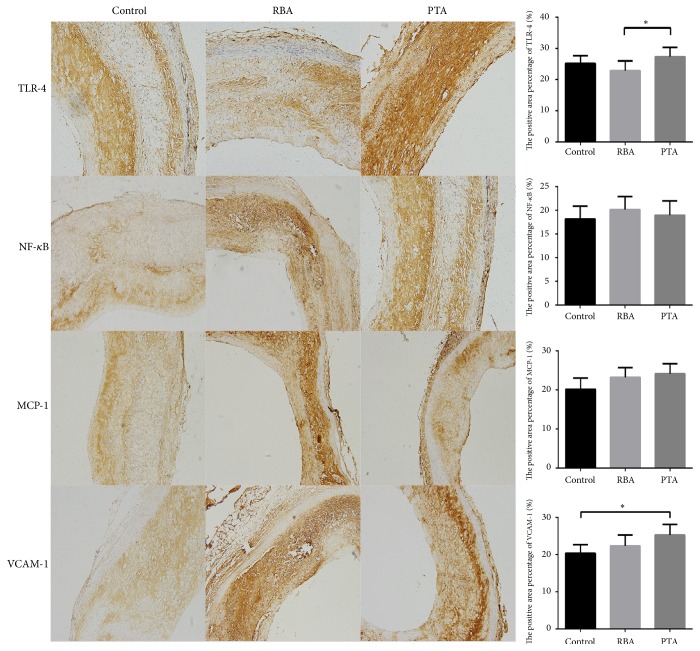
The representative images of immunohistochemistry at 28 days in each group (n=5 in each group; *∗P*<0.05).

Representative images of immunohistochemical staining at 28 days from each group are shown in Figure 5. All atherosclerotic aorta segments showed significant increases of TLR4, NF-*κ*B, MCP-1, and VCAM-1, but their expression was close to zero in the nonatherosclerotic aorta.

**Figure 6:**
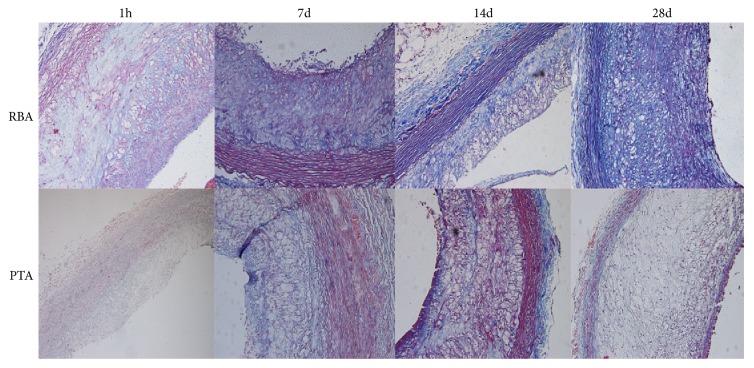
The representative images of Masson trichrome stain in each group.

Representative images of Masson trichrome staining from each group are shown in Figure 6. The specimens from the RBA demonstrated straightening and compression of elastic tissue in the medial aspect of the injury site.

**Figure 7:**
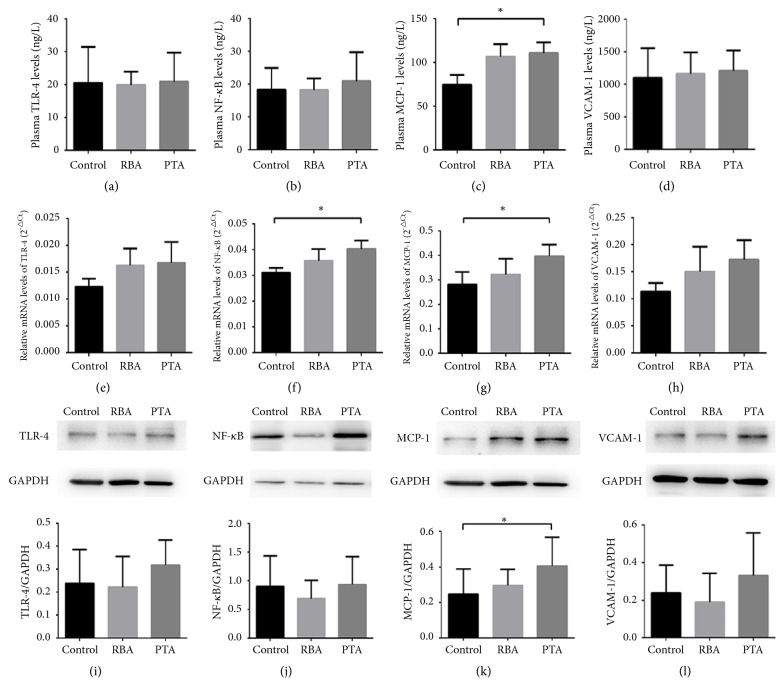
The expression of TLR4, NF-*κ*B, MCP-1, and VCAM-1 in the arteries at 28 days (n=5 in each group; *∗P*<0.05).

Plasma levels of TLR4, NF-*κ*B, MCP-1, and VCAM-1 at 28 days are shown in Figures 7(a), 7(b), 7(c), and 7(d), respectively. TLR4, NF-*κ*B, and VCAM-1 levels were not significantly different among the three groups. By contrast, MCP-1 levels were higher in the PTA group when compared to controls (Figure 7(c);* P*<0.05) whereas no significant differences between RBA and controls were observed.

Relative mRNA expression levels of TLR-4, NF-*κ*B, MCP-1, and VCAM-1 are shown in Figures 7(e), 7(f), 7(g), and 7(h), respectively. TLR4 or VCAM-1 levels were similar across the three groups (*P*>0.05). By contrast, higher NF-*κ*B and MCP-1 were observed PTA group when compared to the controls (*P*<0.05), whereas no significant difference was found between RBA and controls (*P*>0.05).

Finally, protein expression levels of the same proteins detailed above are shown in Figures 7(i), 7(j), 7(k), and 7(l), respectively. No significant difference was observed for TLR-4, NF-*κ*B, or VCAM-1 among the three groups, whereas MCP-1 was higher in PTA group when compared to controls.

## 4. Discussion

The main findings of this study are that (1) aortas subject to high power of RF energy were fragile and (2) when compared to percutaneous transluminal angioplasty, radiofrequency balloon angioplasty produces less intimal hyperplasia but (3) equal increases in vessel size. (4) TLR-4 signaling pathway was not activated, suggesting that it played a minimal role in restenosis.

Consistent with our results, previous experiments have found that thermal treatment attenuates neointimal thickening with enhanced expression of heat-shock protein 72 and suppression of oxidative stress [16–18]. Our results contrast with those of previous studies which displayed greater outward expansion by combined delivery of radiofrequency energy and balloon pressure [2, 3]. The result might be due to lower temperature compared with previous studies.

Animal models are useful system for studying the mechanisms underlying atherosclerosis [19, 20]. Our study and previous animal studies [16, 18] have shown that RBA or thermal treatment can reduce the plaque size, which is a key step in restenosis following balloon angioplasty. This is due to vascular smooth muscle cell (VSMC) proliferation and migration. Atherogenesis is dependent on the innate immune response involving activation of TLRs and the expression of inflammatory proteins [8]. TLR2 and TLR4, subfamilies of TLRs, expressed in lesions, macrophages, dendritic cells, endothelial cells, and vascular smooth muscle cells, have been implicated in the pathogenesis of atherosclerosis [10, 21–23]. TLR-4 may play an more important role in progression of atheromatous plaque to vulnerable plaque causing ACS [24]. Moreover, TLR4 is a vital receptor in arterial remodeling which may leading to restenosis [25]. Activation of TLR-4 induces NF-*κ*B and subsequent transcription of VCAM-1, which are essential for inflammation progression. MCP-1 released from VSMCs, fibroblasts, and macrophages is a major chemokine that induces monocyte or macrophage infiltration. It promotes neointimal formation in early arterial injury lesions [13, 26]. The relationship between TLR4 signal pathway and RBA has not yet been established. The present study demonstrated TLR-4 levels were not significantly increased in either the RBA or PTA groups. This would suggest another signaling pathway by which intimal hyperplasia is mediated. Nevertheless, our findings showed that RBA could ablate medial which represented to straightening and compression of elastic tissue, hyperplasia of collagen fibers, and reduction of cell content. This may in turn lead to a decrease in VSMC proliferation and migration, finally reducing intimal hyperplasia.

Meanwhile, VSMC proliferation and migration have been implicated in a variety of mechanisms. Matrix metalloproteinases (MMPs) have very important roles in neointimal hyperplasia, which develops after vascular injury. Proliferation of VSMC can be promoted by platelet-derived growth factor (PDGF) through upregulating MMP-2 expression, and migration of VSMC can be promoted by Tumor necrosis factor-*α* (TNF-*α*) through upregulating MMP-9 expression, both of which contributed to neointimal hyperplasia [27]. SMC migration caused by arterial balloon injury can be attenuated in the rat model by the transmural expression of the tissue inhibitors of metalloproteinase 1(TIMP-1) by adenoviral gene transfer [28]. Cysteinyl cathepsins also associate with neointimal hyperplasia and VSMC proliferation and migration. Atherosclerotic lesions contain much higher levels of cathepsin K and cathepsin S mRNAs and proteins than normal arteries [29]. Neointimal lesions induced by balloon injury contained significantly higher levels of cathepsin K and cathepsin S mRNAs and proteins than did control arteries [30]. However, whether RBA or thermal treatment can also induce increased expression of MMPs and cathepsins still needs further confirmation.

In contrast to animal studies, clinical data suggest that higher restenosis rate in RBA compared with PTA [5, 6]. This may be due to a number of mechansims being responsible for restenosis. In the early 1980s, intimal hyperplasia was thought to be the primary mechanism by which this occurs. Later studies have found that arterial remodeling is a major determinant of loss of luminal area [31, 32]. To reduce in-stent re-stenosis, strategies such as moderate heating (50°C) by RBA have demonstrated to be a promising approach in reducing cellular proliferation [17]. Moreover, arterial remodeling was found to be more important than neointimal formation in late luminal narrowing after PTA [33, 34]. In support of this notion, the external elastic lamina area (i.e. vessel area), and to a lesser extent intimal thickening, were the most important predictors of luminal area [34].

Furthermore, increased collagen synthesis and deposition contributes to both restenosis and arterial remodeling after PTA [35]. This phenomenon also occurs in pulmonary vein (PV) stenosis post RFCA for atrial fibrillation. For example, the effects of extensive radiofrequency energy applications on the structures of the PVs were examined in dogs [36]. A number of pathological changes including intimal proliferation, necrotic myocardium in various stages of collagen replacement, endovascular contraction, and proliferation of elastic lamina were observed. Vessel wall contraction was observed in PV with luminal narrowing, typically at the site at which RF energy application produced necrosis and consequent scar formation. In the present study, similar processes may be responsible for RF-induced excessive collagen reaction. The early strategy of ablation in PV has shown high rate of PV stenosis [37, 38]. The addition of linear ablation on top of PV isolation decreased the development of PV stenosis [39, 40]. For this study, we designed an intravascular linear ablation catheter aimed to decreased restenosis.

## 5. Limitations

Several limitations of our study should be noted. Firstly, angiography after the ablation was not performed, vasospasm and dissection induced by RF energy may not have been identified. Secondly, assessment of restenosis was not suitable by Hematoxylin and Eosin staining because of shrinkage of the arteries from dehydration. Further studies could use IVUS or OCT as complementary methods. Nevertheless, this is a proof-of-concept study with the aim of producing a prototype of the catheter, demonstrating it to be a viable option for treating atherosclerosis.

## 6. Conclusion

RBA can depress the intimal hyperplasia and promote dilatation of the artery to greater extents than PTA at 28 days. However, this did not involve TLR-4 signaling pathway, which likely plays a negligible role in mediating restenosis. Reduction of intimal hyperplasia may be due to injury of ablation to the tunica media and inhibition of VSMC proliferation and migration.

## Figures and Tables

**Table 1:** 

	**RBA**				**PTA**				**Control**
	**1 hour **(n=5)	**7 days **(n=5)	**14 days **(n=5)	**28 days **(n=5)	**1 hour **(n=5)	**7 days **(n=5)	**14 days **(n=5)	**28 days **(n=5)	**28 days **(n=5)
**Intimal area(mm** ^**2**^ **)**	3.4±1.1	4.0±1.0	3.6±1.2	2.4±0.8	3.8±1.0	1.1±0.1	3.0±1.0	2.9±0.8	3.4±0.6
**Vessel size(mm** ^**2**^ **)**	5.7±1.4	7.1±1.5	5.6±1.4	5.4±1.3	6.6±1.1	7.7±1.8	5.6±1.2	5.7±1.2	5.5±1.8
**Medial area(mm** ^**2**^ **)**	0.9±0.1	1.0±0.2	0.9±0.3	0.8±0.1	1.1±0.1	1.0±0.1	0.8±0.1	0.8±0.1	1.0±0.3
**Luminal area(mm** ^**2**^ **)**	1.5±0.7	2.4±0.7	2.5±0.8	2.2±0.7	2.1±0.5	3.1±1.2	1.9±0.4	2.1±0.7	1.7±0.4
**Ratios of the intima to the media (100**%**)**	4.0±1.6	4.4±1.6	3.9±1.6	3.5±1.8	3.6±1.2	3.7±0.7	3.8±1.5	4.1±1.3	3.9±1.6

## Data Availability

The data used to support the findings of this study are included within the article.

## References

[1] Byrne R. A., Joner M., Kastrati A. (2015). Stent thrombosis and restenosis: what have we learned and where are we going? The Andreas Grüntzig Lecture ESC 2014. *European Heart Journal*.

[2] Lee B. I., Becker G. J., Waller B. F. (1989). Thermal compression and molding of atherosclerotic vascular tissue with use of radiofrequency energy: Implications for radiofrequency balloon angioplasty. *Journal of the American College of Cardiology*.

[3] Fram D. B., Gillam L. D., Aretz T. A. (1993). Low pressure radiofrequency balloon angioplasty: Evaluation in porcine peripheral arteries. *Journal of the American College of Cardiology*.

[4] Kaplan J., Barry K. J., Connolly R. J. (1993). Healing after arterial dilatation with radiofrequency thermal and nonthermal balloon angioplasty systems. *Journal of Investigative Surgery*.

[5] Saito S., Arai H., Kim K., Aoki N. (1994). Initial clinical experiences with rescue unipolar radiofrequency thermal balloon angioplasty after abrupt or threatened vessel closure complicating elective conventional balloon coronary angioplasty. *Journal of the American College of Cardiology*.

[6] Yamashita K., Satake S., Ohira H., Ohtomo K. (1994). Radiofrequency thermal balloon coronary angioplasty: A new device for successful percutaneous transluminal coronary angioplasty. *Journal of the American College of Cardiology*.

[7] Poltorak A., He X., Smirnova I. (1998). Defective LPS signaling in C3H/HeJ and C57BL/10ScCr mice: mutations in *Tlr4* gene. *Science*.

[8] Lin J., Kakkar V., Lu X. (2016). Essential roles of toll-like receptors in atherosclerosis. *Current Medicinal Chemistry*.

[9] Tang Y. L., Jiang J. H., Wang S. (2015). TLR4/NF-*κ*B Signaling Contributes to Chronic Unpredictable Mild Stress-Induced Atherosclerosis in ApoE-/- Mice. *PLoS ONE*.

[10] Falck-Hansen M., Kassiteridi C., Monaco C. (2013). Toll-like receptors in atherosclerosis. *International Journal of Molecular Sciences*.

[11] Jia S.-J., Niu P.-P., Cong J.-Z., Zhang B.-K., Zhao M. (2014). TLR4 signaling: A potential therapeutic target in ischemic coronary artery disease. *International Immunopharmacology*.

[12] Liu M., Yu P., Jiang H. (2017). The Essential Role of Pin1 via NF-*κ*B Signaling in Vascular Inflammation and Atherosclerosis in ApoE-/- Mice. *International Journal of Molecular Sciences*.

[13] Bhat O. M., Uday Kumar P., Harishankar N., Ravichandaran L., Bhatia A., Dhawan V. (2017). Interleukin-18-induced cell adhesion molecule expression is associated with feedback regulation by PPAR-*γ* and NF-*κ*B in Apo E−/− mice. *Molecular and Cellular Biochemistry*.

[14] Yin Y. W., Liao S. Q., Zhang M. J. (2014). TLR4-mediated inflammation promotes foam cell formation of vascular smooth muscle cell by upregulating ACAT1 expression. *Cell Death & Disease*.

[15] Ye X., Jiang X., Guo W., Clark K., Gao Z. (2013). Overexpression of NF-*κ*B p65 in macrophages ameliorates atherosclerosis in apoE-knockout mice. *American Journal of Physiology-Endocrinology and Metabolism*.

[16] Okada M., Hasebe N., Aizawa Y., Izawa K., Kawabe J.-I., Kikuchi K. (2004). Thermal Treatment Attenuates Neointimal Thickening with Enhanced Expression of Heat-Shock Protein 72 and Suppression of Oxidative Stress. *Circulation*.

[17] Brasselet C., Durand E., Addad F. (2008). Effect of local heating on restenosis and in-stent neointimal hyperplasia in the atherosclerotic rabbit model: A dose-ranging study. *European Heart Journal*.

[18] Ellenbroek G. H. J. M., van Hout G. P. J., de Jager S. C. A. (2017). Radiofrequency Ablation of the Atherosclerotic Plaque: a Proof of Concept Study in an Atherosclerotic Model. *Journal of Cardiovascular Translational Research*.

[19] Lee Y. T., Laxton V., Lin H. Y. (2017). Animal models of atherosclerosis. *Biomedical Reports*.

[20] Lee Y. T., Lin H. Y., Chan Y. W. (2017). Mouse models of atherosclerosis: a historical perspective and recent advances. *Lipids in Health and Disease*.

[21] Shuang C., Wong M. H., Schulte D. J., Arditi M., Michelsen K. S. (2007). Differential expression of Toll-like receptor 2 (TLR2) and responses to TLR2 ligands between human and murine vascular endothelial cells. *Journal of Endotoxin Research*.

[22] Akashi S., Shimazu R., Ogata H. (2000). Cutting edge: cell surface expression and lipopolysaccharide signaling via the Toll-like receptor 4-MD-2 complex on mouse peritoneal macrophages. *The Journal of Immunology*.

[23] Biragyn A., Ruffini P. A., Leifer C. A. (2002). Toll-like receptor 4-dependent activation of dendritic cells by *β*-defensin 2. *Science*.

[24] Satoh S., Yada R., Inoue H. (2016). Toll-like receptor-4 is upregulated in plaque debris of patients with acute coronary syndrome more than Toll-like receptor-2. *Heart and Vessels*.

[25] Hollestelle S. C. G., De Vries M. R., Van Keulen J. K. (2004). Toll-like receptor 4 is involved in outward arterial remodeling. *Circulation*.

[26] Lee G.-L., Wu J.-Y., Tsai C.-S. (2016). TLR4-activated MAPK-IL-6 axis regulates vascular smooth muscle cell function. *International Journal of Molecular Sciences*.

[27] Guo L., Ning W., Tan Z., Gong Z., Li X. (2014). Mechanism of matrix metalloproteinase axis-induced neointimal growth. *Journal of Molecular and Cellular Cardiology*.

[28] Dollery C. M., Humphries S. E., McClelland A., Latchman D. S., McEwan J. R. (1999). Expression of tissue inhibitor of matrix metalloproteinases 1 by use of an adenoviral vector inhibits smooth muscle cell migration and reduces neointimal hyperplasia in the rat model of vascular balloon injury. *Circulation*.

[29] Sukhova G. K., Shi G.-P., Simon D. I., Chapman H. A., Libby P. (1998). Expression of the elastolytic cathepsins S and K in human atheroma and regulation of their production in smooth muscle cells. *The Journal of Clinical Investigation*.

[30] Cheng X. W., Kuzuya M., Sasaki T. (2004). Increased Expression of Elastolytic Cysteine Proteases, Cathepsins S and K, in the Neointima of Balloon-Injured Rat Carotid Arteries. *The American Journal of Pathology*.

[31] Andersen H. R., Mæng M., Thorwest M., Falk E. (1996). Remodeling Rather Than Neointimal Formation Explains Luminal Narrowing after Deep Vessel Wall Injury: Insights from a Porcine Coronary (Re) stenosis Model. *Circulation*.

[32] Mintz G. S., Popma J. J., Pichard A. D. (1996). Arterial Remodeling after Coronary Angioplasty: A Serial Intravascular Ultrasound Study. *Circulation*.

[33] Tanaka L. Y., Laurindo F. R. M. (2017). Vascular remodeling: A redox-modulated mechanism of vessel caliber regulation. *Free Radical Biology & Medicine*.

[34] Guzman L. A., Mick M. J., Arnold A. M., Forudi F., Whitlow P. L. (1996). Role of Intimal Hyperplasia and Arterial Remodeling after Balloon Angioplasty: An Experimental Study in the Atherosclerotic Rabbit Model. *Arteriosclerosis, Thrombosis, and Vascular Biology*.

[35] Ota R., Kurihara C., Tsou T. L. (2009). Roles of matrix metalloproteinases in flow-induced outward vascular remodeling. *Journal of Cerebral Blood Flow & Metabolism*.

[36] Taylor G. W., Kay G. N., Zheng X., Bishop S., Ideker R. E. (2000). Pathological effects of extensive radiofrequency energy applications in the pulmonary veins in dogs. *Circulation*.

[37] Fisher J. D., Spinelli M. A., Mookherjee D., Krumerman A. K., Palma E. C. (2006). Atrial fibrillation ablation: reaching the mainstream. *PACE—Pacing and Clinical Electrophysiology*.

[38] Arentz T., Jander N., von Rosenthal J. (2003). Incidence of pulmonary vein stenosis 2 years after radiofrequency catheter ablation of refractory atrial fibrillation. *European Heart Journal*.

[39] Zhang Z., Letsas K. P., Zhang N. (2016). Linear Ablation Following Pulmonary Vein Isolation in Patients with Atrial Fibrillation: A Meta-Analysis. *Pacing and Clinical Electrophysiology*.

[40] Arbelo E., Guiu E., Bisbal F. (2014). Benefit of left atrial roof linear ablation in paroxysmal atrial fibrillation: A prospective, randomized study. *Journal of the American Heart Association*.

